# Instrumented assessment of gait disturbance in PMM2-CDG adults: a feasibility analysis

**DOI:** 10.1186/s13023-024-03027-x

**Published:** 2024-02-02

**Authors:** Lara Cirnigliaro, Fabio Pettinato, Maria Stella Valle, Antonino Casabona, Agata Fiumara, Michele Vecchio, Valerio Amico, Renata Rizzo, Jaak Jaeken, Rita Barone, Matteo Cioni

**Affiliations:** 1 Child Neurology and Psychiatry Unit, Department of Clinical and Experimental Medicine, University of Catania – Policlinico, Via Santa Sofia, 78, 95123 Catania, Italy; 2 Laboratory of Neuro-Biomechanics, Department of Biomedical and Biotechnological Sciences, School of Medicine, University of Catania, Catania, Italy; 3 Referral Centre for Inherited Metabolic Diseases, Department of Clinical and Experimental Medicine, University of Catania, Catania, Italy; 4 Department of Biomedical and Biotechnological Sciences, Section of Pharmacology, University of Catania, 95123 Catania, Italy; 5 Rehabilitation Unit, AOU Policlinico-San Marco, 95123 Catania, Italy; 6 Department of Development and Regeneration, Centre for Metabolic Diseases, University Hospital Gasthuisberg, KU Leuven, Leuven, Belgium; 7 Reseach Unit of Rare Diseases and Neurodevelopmental Disorders, Oasi Research Institute-IRCCS, Troina, Italy

**Keywords:** PMM2-CDG, Ataxia, Gait, Instrumented gait analysis

## Abstract

**Background:**

Congenital disorders of glycosylation (CDG) are genetic diseases caused by impaired synthesis of glycan moieties linked to glycoconjugates. Phosphomannomutase 2 deficiency (PMM2-CDG), the most frequent CDG, is characterized by prominent neurological involvement. Gait disturbance is a major cause of functional disability in patients with PMM2-CDG. However, no specific gait assessment for PMM2-CDG is available. This study analyses gait-related parameters in PMM2-CDG patients using a standardized clinical assessment and instrumented gait analysis (IGA).

**Results:**

Seven adult patients with a molecular diagnosis of PMM2-CDG were followed-up from February 2021 to December 2022 and compared to a group of healthy control (HC) subjects, matched for age and sex. Standardized assessment of disease severity including ataxia and peripheral neuropathy along with isometric muscle strength and echo-biometry measurements at lower limbs were performed. IGA spatiotemporal parameters were obtained by means of a wearable sensor in basal conditions. PMM2-CDG patients displayed lower gait speed, stride length, cadence and symmetry index, compared to HC. Significant correlations were found among the used clinical scales and between disease severity (NCRS) scores and the gait speed measured by IGA. Variable reduction of knee extension strength and a significant decrease of lower limb muscle thickness with conserved echo intensity were found in PMM2-CDG compared to HC.

**Conclusions:**

The study elucidates different components of gait disturbance in PMM2-CDG patients and shows advantages of using wearable sensor-based IGA in this frame. IGA parameters may potentially serve as quantitative measures for follow-up or outcome quantification in PMM2-CDG.

## Introduction

Congenital disorders of glycosylation (CDG) are a heterogeneous group of genetic disorders caused by variants in over 160 genes coding for proteins involved in glycoprotein and glycolipid glycosylation [1]. The most frequently affected pathways are the N-linked and O-linked protein glycosylation, which consist in the covalent linkage of glycan chains to proteins at their glycosylation sites formed by asparagine or serine/threonine residues, respectively [2]. The majority of CDG are multisystem diseases with mostly central nervous system involvement.

Deficiency of phosphomannomutase (PMM) enzyme (*PMM2* mutations), catalysing the second step of the N-glycosylation pathway, causes the most frequent CDG, PMM2-CDG (OMIM 212065), with an estimated incidence of 1:20000 [3, 4]. It is a multisystem disease with a broad neurological phenotype, characterized by prominent cerebellar hypotrophy/atrophy, psychomotor delay/intellectual disability, hypotonia, acquired microcephaly, retinitis pigmentosa, strabismus, epilepsy, stroke-like episodes, hyperkinetic movement disorders and peripheral neuropathy. Extraneurological manifestations, such as failure to thrive, liver dysfunction, pericardial effusion, coagulation disorders, skeletal deformities, hypogonadism, occur to a variable extent.

Cerebellar syndrome is the most constant neurological feature including early, unusual oculomotor disturbances, dysmetria, dysarthria and ataxia [5, 6]. Gait disturbance is one of the most significant causes of functional disability in PMM2-CDG [7–9]. However, no instrumented gait characterization of PMM2-CDG patients has been described so far.

Clinical trials and potential therapies for CDG are finally emerging [10–14], strengthening the importance to develop sensitive and objective biomarkers/outcome measures. Recent advances in wearable sensor accuracy technology have made possible the instrumented gait analysis (IGA) as an easy-to-use quantitative assessment tool in the clinical setting supporting diagnosis, treatment evaluation and functional rehabilitation [15, 16].

This study was undertaken to deepen our current understanding of gait-related functional impairment in PMM2-CDG patients, using standardized clinical measures along with biomechanical evaluations and instrumented gait analysis. We foresee that a set of gait-related outcome parameters might be beneficial in clinical trials and in clinical decision-making, enabling an instrumented approach in the evaluation and follow-up of gait disturbance in PMM2-CDG.

## Methods

### Participants

Seven subjects with a molecular diagnosis of PMM2-CDG were included in the study and compared to a group of healthy control (HC) subjects, recruited among acquaintances and matched to PMM2-CDG patients by age (yrs), weight (W) and height (H) (age: patients 37 ± 11.6, HC 37.5 ± 15.1, p = 0.95; W (kg): patients 53.6 ± 15.7, HC 62.63 ± 5.82, p = 0.21; H (cm): patients 153.8 ± 12.1, HC 162.7 ± 5.5, p = 0.13]. The control group was free of diabetes and had no existing medical problems limiting physical activity. Patients were followed-up at the Child Neuropsychiatry Unit, University Hospital of Catania, Italy, from February 2021 to December 2022. Exclusion criteria were the presence of inability to walk, severe intellectual disability, no compliance with gait functional evaluation, sensory defects and a history or evidence of gait disorders not related to the underlying genetic disease.

Clinical history was collected and each patient underwent physical and neurological examination, with emphasis on the assessment of cerebellar symptoms, evaluation of lower limb muscle strength and peripheral neuropathy. Cognitive functioning and the presence of epilepsy and stroke-like episodes were carefully investigated as part of the routine clinical evaluation prior to the instrumental assessment.

Brain magnetic resonance biometry of the cerebellum and brainstem was performed to evaluate the atrophic changes documented in PMM2-CDG patients.

PMM2 variants were classified as mild (missense variants) or severe (loss-of-function variants) following careful analysis of the literature on genotype–phenotype correlation [4, 17].

### Standardized measurements

The severity of neurological involvement, including ataxia, and adaptive functioning were assessed through validated tools such as the Nijmegen CDG Rating Scale (NCRS), the Brief Ataxia rating scale (BARS) and by the use of the activities of daily living (ADL) scale, respectively.

The NCRS is a tool specifically developed to assess clinical manifestations and disease progression of CDG patients. It includes three sections (I-III): current function, system-specific involvement and current clinical assessment [18]. We collected information from all sections with special regard to the current clinical assessment (section III): a careful neurological examination allowed to search for cerebellar, pyramidal, extrapyramidal symptoms, myopathy and neuropathy. Patients and caregivers were also interviewed about vision, hearing, communication and feeding difficulties, mobility and educational achievements. A score was assigned to each item, reflecting normal (0), mild (1), moderate (2) and severe (3) impairment. NCRS global scores refer to mild (0–14), moderate (15–25) and severe (> 26) disease. The total and section III scores of the NCRS were used for correlation analyses.

The BARS was developed to assess cerebellar symptoms in the clinical settings. It is structured on 5 major domains: gait, upper and lower limb coordination, speech and ocular coordination [19]. Total score (maximum score of 30) is related to the evaluation of gait (score range 0–8), knee-tibia test and finger-to-nose test (maximum score of 4 for each limb), dysarthria (score range 0–4) and oculomotor abnormalities (score range 0–2). The gait score of the BARS (item-subset A) along with the knee-tibia test score (item-subset B) and the finger-to-nose test score (item-subset C) for lower and upper limb coordination respectively, were used for correlation analyses, in addition to the BARS total score.

Information on adaptive functioning was gathered using the ADL with focus on walking capacity and movement autonomy, personal care skills and communication abilities. Data were collected after an exhaustive interview with the primary caregiver. Scores ranged from 0 (normal function) to 4 (severe functional disability), with a maximum overall score of 36, indicating very severe functional disability [20].

### Isometric muscle strength and muscle ultrasound examinations

Isometric muscle strength at knee extensor muscles was measured by a hand-held dynamometer (Citec, Holland). Participants were asked to sit with hips and knee flexed to 90º. The resistance exerted by the examiner was applied just proximally to the malleoli. Shoulders stabilization was provided.

Ultrasound muscle evaluation was performed using a 6–15 MHz linear matrix probe (GE LOGIQTM E10, GE Healthcare, Italy). In each patient the rectus femoris (RF), the tibialis anterior (TA), the gastrocnemius medialis (GM) and the gastrocnemius lateralis (GL) were studied bilaterally. The ultrasound examination was carried out in two-dimensional mode (B-Mode), which allowed to measure some anatomical parameters of the RF, TA, GM, GL muscles, including muscle and subcutaneous tissue diameters and their ratio. Furthermore, muscle cross-sectional areas (mCSA) of the RF and TA muscles were measured. Muscle quality was assessed at the level of the middle third of the RF and TA muscles, using an elastographic evaluation by Shear Wave over 3 ROIs, with the analysis of the gray scale and the Echo level.

### Instrumented gait analysis

Instrumented gait analysis (IGA) was performed by means of a single wearable sensor, composed by an accelerometer, a gyroscope and a magnetometer (G-Sensor, BTS, Garbagnate Milanese, Italy), applied over the first sacral vertebra. The sensor was connected via Bluetooth with a server processing the entering data through a specific commercial software (G-Studio, BTS Garbagnate Milanese, Italy). Validated pattern recognition algorithms, previously adopted in patients with ataxia, were used to measure gait-related spatiotemporal parameters and symmetry index [16]. We selected those measures that were considered as candidate promising parameters in cerebellar ataxia based on previous studies [7, 21]: gait speed (walking speed in the designated direction), stride length (distance between two sequential points of ground contact of the same foot), swing phase (part of the gait cycle during which the foot has no contact with the ground), stance phase (part of the gait cycle during which the foot has contact with the ground) and cadence (number of steps/minute). To estimate the symmetry index, we used the correlation analysis on data captured over the right and left gait cycle. Then, from the raw acceleration signals, we extracted and normalised the anterior–posterior acceleration.

Participants were instructed to walk along a 10-m straight path, at self-selected comfortable and slow speeds. Each patient walked along the path for three times under each speed, with inter-trial pauses of approximately 5 min. If the participant complained of fatigue, a further break of 5 min was granted until the resumption of suitable conditions.

### Statistical analysis

Dedicated algorithms were used to calculate gait parameters. Data were analysed with non-parametric statistics since the normal distribution was not verified by the Shapiro–Wilk test. Group comparisons were performed by means of Mann–Whitney U test. Gait spatiotemporal parameters were correlated with clinical rating scores in the CDG group by the Pearson's Chi-square independence test. Moreover, the Chi-square test was applied to assess correlations of BARS, NCRS, and ADL total scores with each other.

### Ethical considerations

All the procedures performed in the present study represent the standard clinical care of patients with gait disturbances and are in accordance with the 1964 Declaration of Helsinki and its later amendments (2013). Written informed consent was obtained from all participants and/or their relatives for enrolment in this study.

## Results

Seven adult patients with a genetically confirmed diagnosis of PMM2-CDG (six males and one female; mean age: 37 ± 10.8, age range: 18–49 years) were studied. Demographic, genetic and clinical data of PMM2-CDG patients are reported in Table 1.

**Table 1:** Clinical and molecular characteristics of the studied patients

	Patient 1	Patient 2	Patient 3	Patient 4	Patient 5	Patient 6	Patient 7
Family	F1	F1	F2	F2	F3	F4	F5
Sex	Female	Male	Male	Male	Male	Male	Male
Age (years)	47	49	38	44	18	33	31
Pathogenic variant	R141H/N216I	R141H/N216I	R141H/V129M	R141H/V129M	R141H/N216I	R141H/L32R	R141H/D223N
Phenotype	Severe	Moderate	Severe	Moderate	Moderate	Mild	Severe
Dysmorphic features	+	+	+	+	+	+	+
Motor disability	+	+	+	+	+	−	+
Retinopathy	+	+	+	+	+	+	+
Intellectual disability	Moderate	Moderate	Moderate	Moderate	Moderate	Moderate	Moderate
Cerebellar atrophy	Severe	Severe	Severe	Severe	Severe	Moderate	Severe
Microcephaly	+	+	−	−	+	−	−
Epilepsy	−	−	+	+	−	−	+
Stroke-like episodes	+	+	+	+	−	−	+
Peripheral neuropathy (tibial nerve mCV)	30 m/s	29 m/s	27 m/s	31 m/s	29 m/s	NA	30 m/s
NCRS T	37	30	40	29	27	8	39
NCRS I	13	11	14	10	6	2	13
NCRS II	6	3	4	3	3	1	6
NCRS III	18	16	22	16	18	4	20
BARS T	22	22	22	22	20	6	22
BARS A	8	8	7	7	7	0	8
BARS B	4	4	4	4	4	2	4
BARS C	4	4	4	4	3	2	4
ADL T	24	21	24	21	19	9	24

+ (present); − (absent); mCV: motor conduction velocity; NA: not acquired; NCRS T: Nijmegen CDG Rating Scale Total score mild (0–14), moderate (15–25) and severe (> 26) disease; NCRS I: Current function score; NCRS II: System specific involvement score; NCRS III: Current clinical assessment score; BARS T: Brief ataxia rating scale (BARS) Total score (maximum severity score: 30); BARS A: Gait score; BARS B: Knee-tibia test score; BARS C: Finger-to-nose test score. ADL T: Activity of Daily Living scale Total score (maximum severity score: 36)

The CDG group included two pairs of siblings (patients #1, #2 and #3, #4 respectively). Patients were compound heterozygous for PMM2 variants and they all harboured the common p.R141H variant [17]. A missense variant (p.L32R), resulting in mild protein change with residual activity [5], was carried by the patient with the milder neurological phenotype (#6).

The most common early clinical signs were global developmental delay, severe hypotonia, failure to thrive and strabismus. At the time of the study, all patients had moderate intellectual disability. Three patients (#3, #4 and #7) developed seizures before the age of 10 years and they were still on antiepileptic therapy with good seizure control. Five patients (#1, #2, #3, #4, #7) had stroke-like episodes during the course of the disease without residual neurological deficits at the study time. All patients showed cerebellar ataxia and peripheral neuropathy with areflexia. They were able to sit unsupported and walk on a flat surface with support. Patient #6 (male, aged 33 y) walked unaided. Nerve conduction velocity assessment showed signs of demyelinating peripheral neuropathy with increased distal latencies and reduction of motor nerve conduction velocity (tibial motor nerve conduction velocity: mean 29 m/s ± 1.8, normal values > 41 m/s) (Table 1).

**Fig. 1:**
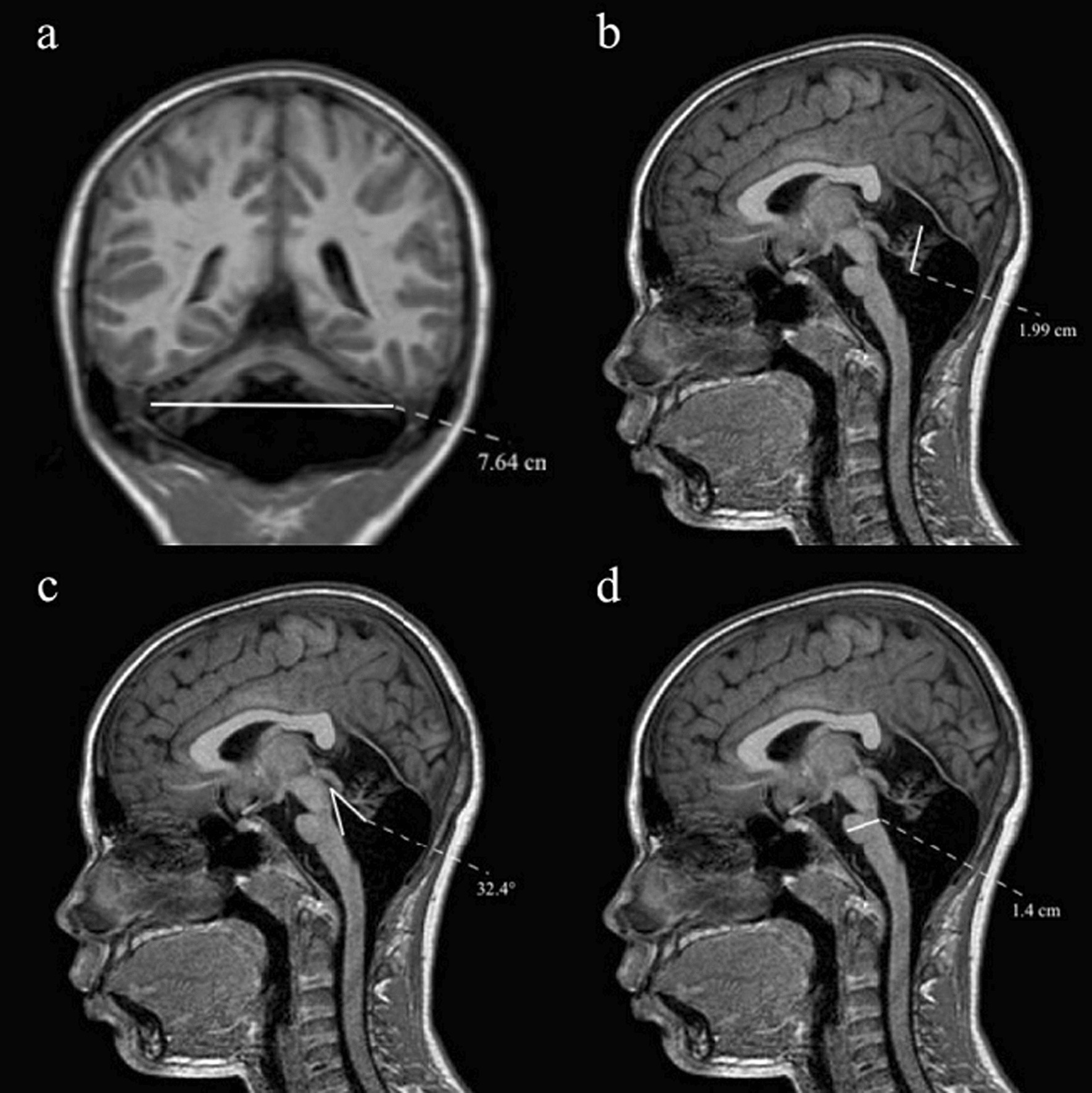
Representative brain MRI biometry (Patient 5). **a** Maximum transverse cerebellar diameter (MCD). **b** Maximum craniocaudal height of the vermis (CCD). **c** Tegmento-vermian angle (TVA). **d** Mid-pons diameter (**a** T1-weighted coronal image; **b**–**d** midline T1-weighted sagittal images)

Cerebellum and brainstem MRI biometry data (cm) were computed (mean ± standard deviation) and compared to internal reference values [8]. They all showed different degrees of cerebellar atrophy on brain MRI. There were atrophic changes of cerebellar hemispheres (maximum transverse cerebellar diameter 7.73 ± 0.17; normal values, n.v. 9.90 ± 0.56) and cerebellar vermis (craniocaudal diameter 1.88 ± 0.58; n.v. 4.43 ± 0.26) with increased measures of the tegmento-vermian angle (25.1 ± 4.14; n.v. 9.88 ± 3.58). Biometry measures of the mid-pons were also decreased (1.14 ± 0.07; n.v. 2.06 ± 0.17) (Fig. 1).

### Standardized measurements

NCRS current clinical assessment (section III), BARS and ADL scores are reported in Table 1. One patient (#6) showed a milder neurological impairment (NCRS section III score = 4 out of 31; BARS Total score = 6 out of 22). Most patients (6 of 7) presented a severe degree of neurological impairment (NCRS section III mean score = 18.3 ± 2.3 out of 31; BARS mean total score = 21.6 ± 0.8 out of 22) with severe functional disability (ADL mean total score = 22.1 ± 2.1 out of 36).

Patients showed ataxic gait with upper/lower limb dysmetria (moderate/severe in six of seven patients). Six patients showed myopathy with symmetrical weakness limiting mobility. They had peripheral neuropathy with decreased osteotendinous reflexes or areflexia and distal muscle atrophy. Extrapyramidal signs included episodic dystonia in all patients. Patient #6 had mild dysmetria, milder neuropathy without muscle atrophy and no myopathy signs.

Higher NCRS and BARS total scores correlated with higher total ADL score (r = 0.92, *p* = 0.009 and r = 0.96, *p* = 0.002 respectively). As a whole, patients with a severe clinical phenotype and more pronounced gait ataxia showed more severe functional disability.

### Instrumental investigations

#### Isometric muscle strength and muscle ultrasound examinations

Five of seven study patients underwent lower limb instrumental muscle strength measurement as two patients (#3 and #7) were not able to push against the dynamometer. Lower limb muscle strength was measured twice, and the higher value was used as absolute knee extension strength (KES) (normal reference values > 160 Nm). Muscle strength reductions were observed in study patients with values ranging from 45 to 80% of lower normal value, with only one subject (#6) having normal KES bilaterally.

**Fig. 2:**
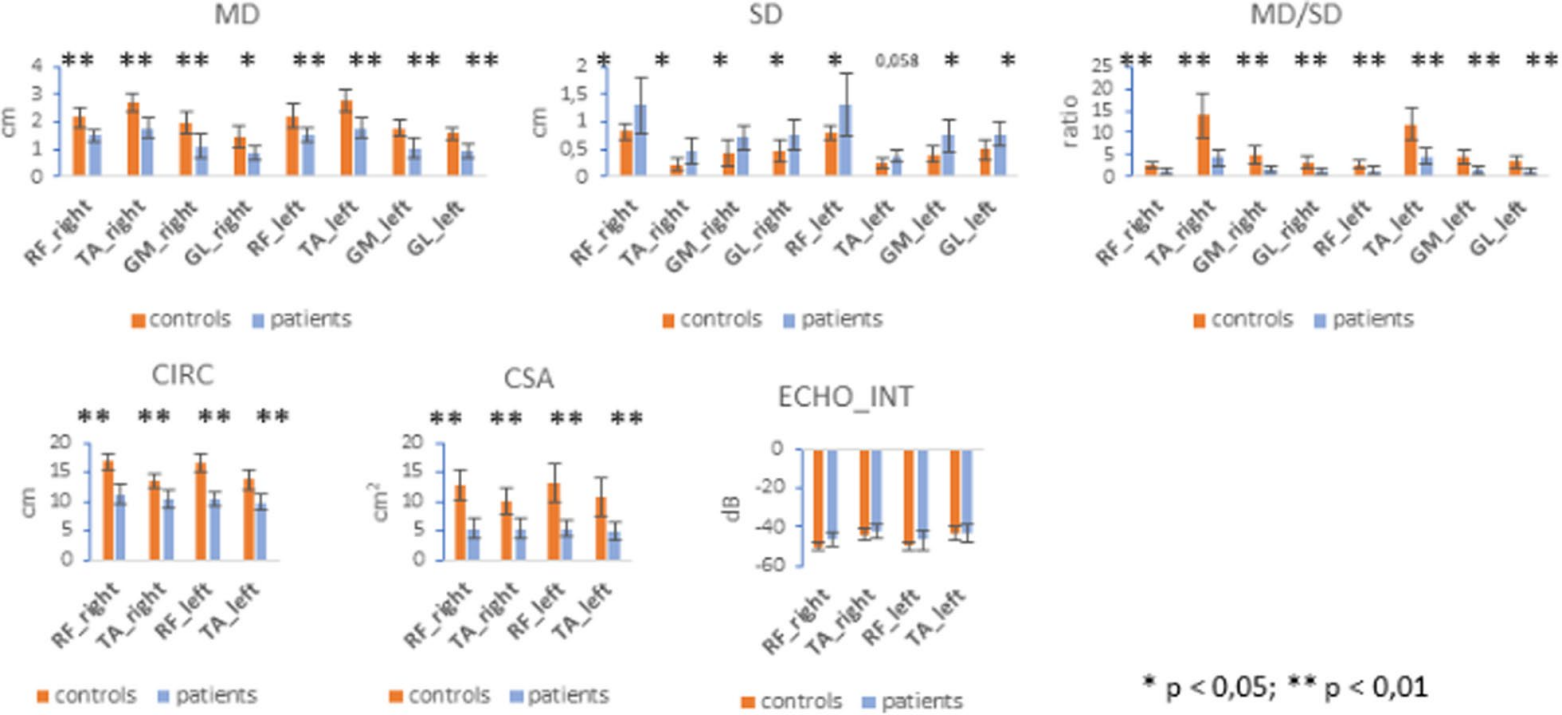
Comparisons among echo parameters of rectus femoris (RF), tibialis anterior (TA), gastrocnemius medialis (GM) and gastrocnemius lateralis (GL) muscles in PMM2-CDG patients and healthy control (HC) group. *RF* rectus femoris muscle; *TA* tibialis anterior muscle; *GM* gastrocnemius medialis muscle; *GL* gastrocnemius lateralis muscle; *MD* muscle diameter; *SD* subcutaneous diameter; *CIRC* circumference; *CSA* cross sectional area; *ECHO* echo intensity

**Fig. 3:**
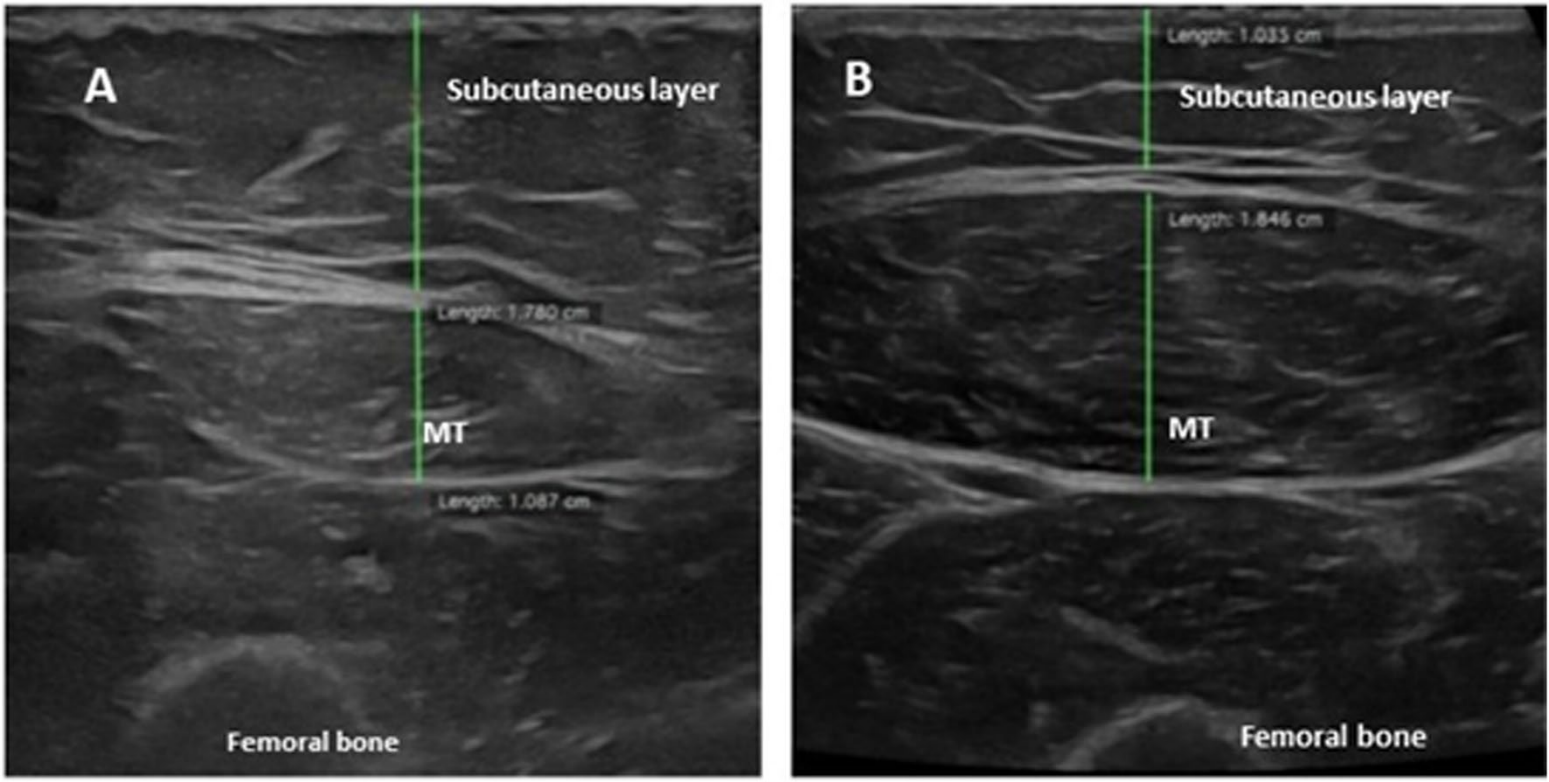
Representative ultrasound images of rectus femoris muscle in PMM2-CDG patient (**A**) compared to age-matched healthy subject (**B**). Muscle thickness (MT) is reduced in favour of increased subcutaneous tissue layer in the patient compared to age and sex matched healthy subject

In order to screen for the presence of muscle quantity and quality changes, PMM2-CDG patients underwent standard B-dimensional lower limb muscular echography at the RF, TA, GM and GL muscles. Significant differences of echo-biometric measures of studied muscles in patients compared to the HC group are depicted in Fig. 2. Muscle diameters were significantly reduced in the patients compared to the HC (i.e., rectus femoris PMM2-CDG 1.50 cm ± 0.25, HC 2.19 cm ± 0.41; p = 0.002). In the group with PMM2-CDG, subcutaneous tissue thickness was increased and the muscle/subcutaneous ratio was significantly reduced compared to the control group (i.e., rectus femoris/subcutaneous ratio: PMM2-CDG 1.30 ± 0.84, HC 2.76 ± 0.62; p = 0.004). Muscle cross sectional areas were significantly reduced in PMM2-CDG patients (i.e., rectus femoris PMM2-CDG 5.32 ± 1.49, HC 12.97 ± 2.95, p < 0.001; tibialis anterior PMM2-CDG 5.05 ± 1.64, HC 10.43 ± 2.79, p = 0.001), indicating a smaller muscle size in the patient group. Echo intensities of RF and TA were within the range of expected normal values (Fig. 3). No correlation was found between TA muscle cross sectional areas and tibial nerve motor conduction velocity in the studied patients (r = 0.45, p = 0.32).

#### Instrumented gait analysis: spatiotemporal parameters

Observational gait analysis showed gait unsteadiness with a large base of support. Most patients showed instability of pelvis in the frontal plane with trunk bending to allow the contralateral foot clearance from the ground. During the swing phase of the gait cycle, the proximal segments of the lower limbs were excessively raised so that the foot came up off the ground, with limited knee flexion. Ground contact occurred with flat foot.

Six of seven study patients underwent instrumented gait analysis as one patient (#3) was unable to fully cooperate. Spatiotemporal gait parameters and statistical comparisons are illustrated in Table 2.

**Table 2:** Spatiotemporal gait parameters in PMM2-CDG patients compared to the healthy control (HC) group

Spatiotemporal gait parameters	A	B	C	D	E
	PMM2-CDG (n = 6)	Controls (n = 6) comfortable speed	Mann–Whitney U (*p*) A vs B	Controls (n = 6) slow speed	Mann–Whitney U (*p*) A vs D
Cadence (step/min)	86.63 ± 8.51	107.32 ± 2.11	**0.025**	82.12 ± 4.50	0.337
Speed (m/s)	0.38 ± 0.06	1.09 ± 0.02	**0.004**	0.69 ± 0.04	**0.008**
Stride length (m)	0.57 ± 0.09	1.23 ± 0.03	**0.004**	1.01 ± 0.06	**0.004**
Left stance duration (% cycle)	59.87 ± 2.71	61 ± 0.75	0.631	59.49 ± 0.81	0.749
Right stance duration (% cycle)	57.5 ± 2.09	60.9 ± 0.44	0.15	61.04 ± 0.45	0.15
Symmetry index	82.82 ± 5.37	96.13 ± 0.74	**0.01**	94.97 ± 1.01	**0.025**

Statistically significant values are reported in bold

**Fig. 4:**
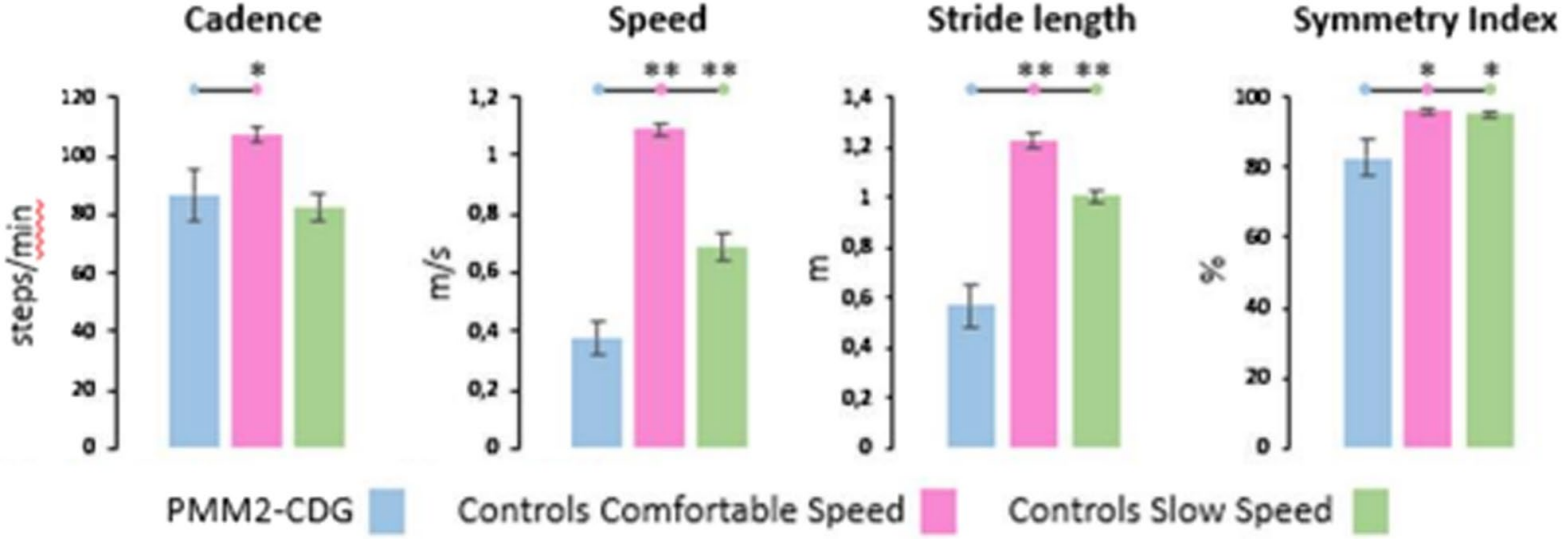
Spatiotemporal gait parameters in PMM2-CDG patients compared to the healthy control (HC) patients walking at comfortable speed and slow speed

PMM2-CDG patients showed a slower average gait speed, in comparison to controls, either at a self-determined comfortable speed (*p* = 0.004) and at low speed (*p* = 0.008). Similarly, PMM2-CDG patients compared to controls had, in both walking speed conditions, significantly decreased stride length (comfortable speed: *p* = 0.004; low speed: *p* = 0.004) and symmetry index (comfortable speed: *p* = 0.01; low speed: *p* = 0.025). Differences in cadence, instead, were evident in patients compared to controls, at a comfortable speed (*p* = 0.025), but not at low speed (*p* = 0.337) (Fig. 4). No differences were found between the two groups in the right and left stance durations.

Correlation analyses showed that reduced gait speed was significantly correlated with higher NCRS section III (current clinical assessment) and total scores (r =  − 0.89, *p* = 0.018 and r =  − 0.81, *p* = 0.050) respectively), demonstrating that the gait speed parameter is not only an indicator of impairment but that it can also be predictive of disease severity.

## Discussion

In the present study we applied clinical and instrumental parameters including isometric muscle strength, nerve conduction velocity, echographic muscle anatomical features and gait spatiotemporal parameters, in order to objectively measure and correlate several biomechanical factors implicated in the gait disturbance of PMM2-CDG patients. Ambulatory function is almost constantly impaired in PMM2-CDG with most patients requiring support or being unable to walk [8, 22, 23]. Ambulatory impairment is a reflection of several causative factors including balance, strength and orthopaedic issues [3]. Cerebellar ataxia has been reported from 96.7% [23] to 100% [5, 6, 24] of PMM2-CDG patients. Different standardized measures have been used to assess ataxia in PMM2-CDG patients, such as the NCRS [18], the International Cooperative Ataxia Rating Scale (ICARS) [24], the Spinocerebellar Degeneration Functional Score (SDFS) [22] and the Brief Ataxia Rating Scale [8]. ICARS scores correlated with NCRS and were higher in patients with severe PMM2 variants and severe cerebellar atrophy [24]. The neurological spectrum of PMM2-CDG also includes non-cerebellar features, such as muscle hypotonia, muscular weakness, peripheral neuropathy, and cognitive impairment which may add to the ambulatory impairment of PMM2-CDG patients. Therefore, multiple clinical and instrumental assessments are potentially needed to collectively illustrate the effects of disease and disability on gait and function in CDG patients. In the current study we showed that wearable sensor-based IGA enables accurate measurement of gait metrics, such as speed, cadence, stride length, stance/swing phase and symmetry index in PMM2-CDG patients. Namely, we found slower average gait speed in PMM2-CDG patients compared to controls walking at comfortable and slow speed. Interestingly, we found significant correlations among all clinical scales used and between the NCRS scores (CDG severity scale) and the gait speed, measured by IGA, supporting a functional interplay among these measures. Thus, the gait speed parameter may be considered as an indicator of functional impairment [25]. We suggest that gait disturbances in PMM2-CDG patients depends on central and peripheral abnormalities. In this study, we documented cerebellar atrophy, mostly vermian atrophy and a remarkable midpons atrophy on MRI biometry. Cortical projections and sensory input from lower limbs to the vermis are believed to be part of the neural network involved in fine tuning of limb motion, anticipatory postural adjustments and locomotion [26, 27]. Possibly due to the small sample size, we could not find a correlation between the MRI findings and IGA spatiotemporal parameters. However, the pattern of gait in cerebellar ataxia we found is consistent with previous findings in ataxic disorders [7] and includes lower average gait speed and stride length and reduced cadence; it might reflect a defective connectivity in the higher control of gait through corticocerebellar projections [28]. Peripheral neuropathy, occurring in almost 60% of PMM2-CDG patients, causes abolished osteotendinous reflexes, distal atrophy and pes planus [4]. It contributes to muscle weakening and atrophy and to impaired motor ability in PMM2-CDG [29], also in the studied patients. Muscle cross-sectional area and thickness, are factors affecting muscle strength [30, 31]. We found significantly reduced muscle diameters and cross section areas in PMM2-CDG patients compared to the control group. This indicates a smaller muscle size in the patient group, resulting in a proportional muscle strength reduction. Myopathy is an underreported feature in PMM2-CDG. However, myopathy signs of variable severity were recently reported in about 80% of 51 patients with PMM2-CDG [32]. In the current study, we failed to demonstrate a direct correlation between nerve conduction velocity and muscle size parameters, suggesting that besides peripheral neuropathy, additional, still unexplored factors might contribute to muscle size reduction in PMM2-CDG.

Motor neuropathy reduces lower extremity muscle size and strength in metabolic diseases, such as type 2 diabetes [31]. In patients with diabetes, treatment with the aldose reductase inhibitor, epalrestat, reduces sorbitol levels and halts neuropathy progression [33]. Elevated urinary excretion of sorbitol, occurring in 74% of PMM2-CDG patients, correlated with disease severity and peripheral neuropathy in these patients. Epalrestat was effective in increasing PMM enzyme activity, ameliorating glycosylation and decreasing sorbitol levels in PMM2-deficient fibroblasts [33, 34]. Moreover, safety and efficacy of epalrestat were studied in a PMM2-CDG patient for 12 months with amelioration of ataxia scores and growth parameters [34]. The present data, showing decreased muscle strength and thickness in PMM2-CDG patients, support the possible effectiveness of epalrestat therapy also in preventing and/or halting the effects of peripheral neuropathy in these patients. Moreover, IGA showed that stride length and symmetry index were significantly reduced in PMM2-CDG patients compared to controls, either at a self-determined comfortable speed and at low walking speed. Differences in cadence, instead, were evident in patients compared to controls, at a comfortable speed, but not at low speed. The control of walking speed depends on two inversely proportional parameters: cadence and stride length [35]. Studied patients increased cadence to increase gait speed possibly due to contractures of proximal lower limb muscles. Then, we can hypothesize that the central neural mechanisms regulating the cadence of stepping may still be activated in these patients. A clinical implication of this finding might be that these patients should receive a more intensive rehabilitation intervention aimed at stretching the hip muscles to increase stride length, in order to achieve a greater functional autonomy.

Main limitation of our study is the focus on a single-center, hospital-based sample of patients with PMM2-CDG enrolled over one year. Nevertheless, all patients were fully characterized by the same physicians using a strict clinical protocol. Albeit the limited sample size, we first show that the use of a single wearable sensor contributes significantly making the measurements of motor tasks operationally easier in PMM2-CDG patients, without neglecting a perfect reliability and the possible application in a home monitoring setting [16]. In recent years, an accurate assessment of the severity of gait impairment has become a prerequisite for clinical trials of novel therapies for cerebellar ataxia syndromes [15]. Additional clinical studies may profit from detailed metrics of the spatiotemporal parameters to get new insight into the nature of gait impairments with the potential to serve as an independent, quantitative clinical biomarker in PMM2-CDG, as has been proposed for other rare diseases [36]. In conclusion, the present study elucidates different components of gait disturbance in PMM2-CDG and shows potential advantages of using wearable sensor-based IGA for outcome quantification.

## Data Availability

The data that support the findings of this study are not openly available due to reasons of sensitivity and are available from the corresponding author upon reasonable request. Data are located in controlled access data storage at the University of Catania.
